# Characterization of *N*-Acyl Phosphatidylethanolamine-Specific Phospholipase-D Isoforms in the Nematode *Caenorhabditis elegans*

**DOI:** 10.1371/journal.pone.0113007

**Published:** 2014-11-25

**Authors:** Neale Harrison, Museer A. Lone, Tiffany K. Kaul, Pedro Reis Rodrigues, Ifedayo Victor Ogungbe, Matthew S. Gill

**Affiliations:** Department of Metabolism & Aging, The Scripps Research Institute, Scripps Florida, Jupiter, Florida, United States of America; Virginia Commonwealth University, United States of America

## Abstract

*N*-acylethanolamines are an important class of lipid signaling molecules found in many species, including the nematode *Caenorhabditis elegans* (*C. elegans*) where they are involved in development and adult lifespan. In mammals, the relative activity of the biosynthetic enzyme *N*-acyl phosphatidylethanolamine-specific phospholipase-D and the hydrolytic enzyme fatty acid amide hydrolase determine *N*-acylethanolamine levels. *C. elegans* has two *N*-acyl phosphatidylethanolamine-specific phospholipase-D orthologs, *nape-1* and *nape-2*, that are likely to have arisen from a gene duplication event. Here, we find that recombinant *C. elegans* NAPE-1 and NAPE-2 are capable of generating *N*-acylethanolamines *in vitro*, confirming their functional conservation. *In vivo*, they exhibit overlapping expression in the pharynx and the nervous system, but are also expressed discretely in these and other tissues, suggesting divergent roles. Indeed, *nape-1* over-expression results in delayed growth and shortened lifespan only at 25°C, while *nape-2* over-expression results in significant larval arrest and increased adult lifespan at 15°C. Interestingly, deletion of the *N*-acylethanolamine degradation enzyme *faah-1* exacerbates *nape-1* over-expression phenotypes, but suppresses the larval arrest phenotype of *nape-2* over-expression, suggesting that *faah-1* is coupled to *nape-2*, but not *nape-1*, in a negative feedback loop. We also find that over-expression of either *nape-1* or *nape-2* significantly enhances recovery from the dauer larval stage in the insulin signaling mutant *daf-2(e1368)*, but only *nape-1* over-expression reduces *daf-2* adult lifespan, consistent with increased levels of the *N*-acylethanolamine eicosapentaenoyl ethanolamine. These results provide evidence that *N*-acylethanolamine biosynthetic enzymes in *C. elegans* have conserved function and suggest a temperature-dependent, functional divergence between the two isoforms.

## Introduction

*N*-acylethanolamines (NAEs) are bioactive lipids that are involved in diverse physiological processes in both animals and plants, and are composed of an acyl chain of varying length and unsaturation that is amide-linked to an ethanolamine head group [1], [2]. In mammals, the first step in the NAE biosynthetic pathway is the synthesis of *N*-acyl phosphatidylethanolamine (NAPE) from phosphatidylethanolamine and phosphatidylcholine, by the enzyme *N*-acyl transferase (NAT) [1]. Although various Ca^2+^ independent isoforms have been cloned and characterized, the Ca^2+^ dependent NAT has yet to be identified [3]. The glycerophospholipid substrates of this enzyme are abundantly found in most membranes, but levels of NAPEs are maintained at very low levels [4], suggesting that the activity of NAT represents the rate-limiting step in NAE biosynthesis. In the second step, NAEs are synthesized from their precursor NAPEs by the enzyme NAPE-specific phospholipase-D (NAPE-PLD) [5], [6]. Cloning of mammalian NAPE-PLD revealed no homology to other known PLDs, but instead indicated that it belongs to the novel zinc metallohydrolase family with a β-lactamase fold [7]. NAPE-PLD is capable of producing various medium and long chain bioactive NAEs in a Ca^2+^-dependent manner [7] and the enzyme is specific for NAPE substrates, with no activity towards other phospholipids [8].

Among other enzymes of the endocannabinoid metabolic pathway, sequence homologs of NAPE-PLD have been identified in many organisms [9], and NAPE-PLD has been cloned and characterized from rat, mouse and humans [7]. Consistent with the widespread function of NAEs, NAPE-PLD expression in mammals is detected in most tissues, being particularly high in brain and testis [7]. Mammalian NAPE-PLD is a membrane bound protein, localized mainly to the intracellular microsomal membranes. Due to the magnitude of physiological responses to minute changes in levels of different NAEs at the tissue and organismal level, the activity of NAPE-PLD is tightly controlled, especially at the transcriptional level [10], [11]. The enzyme activity is stimulated by Ca^2+^ and activation of ionotropic glutamate N-methyl-D-aspartate receptors [12], as well as by the major neurotransmitters, dopamine [13], glutamine [14] and acetylcholine [15]. In the last step of the pathway, NAE levels are then regulated by fatty acid amide hydrolase (FAAH) which hydrolyses NAEs into free fatty acids and ethanolamine [16]. Though the levels of NAEs are mostly regulated through this canonical pathway, other anabolic and catabolic enzymes have been described that modulate NAE levels [3].

Previous work from our laboratory identified the enzyme components for NAE metabolism in the free living nematode, *C. elegans*, as well as a set of diverse NAEs that differed in their fatty acid composition and level of unsaturation [17]. While we were able to show that NAE levels were modulated by over-expression of the *C. elegans* homolog of FAAH, we did not address the role of NAE biosynthetic enzymes in the worm, other than to show that one of the orthologs, *nape-1*, was expressed in similar tissues to *faah-1* [17]. In the present work we have carried out a more detailed examination of the two *C. elegans* homologs of the NAE synthesizing enzyme NAPE-PLD, *nape-1* and *nape-2*. We show that both proteins are capable of catalyzing the production of NAEs *in vitro*, indicating that they are functional orthologs of mammalian NAPE-PLD. We also show that despite strong sequence similarity, the function of the two homologs may have diverged and they may be responsible for temperature-dependent effects of NAEs on development and lifespan.

## Materials and Methods

### Chemicals

Palmitoyl ethanolamide-*d4* (PEA-*d4*) and arachidonoyl ethanolamide-*d4* (AEA-*d4*) were obtained from Cayman Chemical (MI). *N*-palmitoyl phosphatidylethanolamine (*N*-palmitoyl PE) was obtained from Enzo Life Sciences (PA) and *N*-arachidonoyl PE (1,2-dioleoyl-*sn*-glycero-3-phosphoethanolamine-*N*-arachidonoyl ammonium salt) was obtained from Avanti Polar Lipids, Inc (AL). BSTFA was from Sigma Aldrich (MO). All solvents were of gas chromatography-mass spectrometry (GC-MS) grade and all other reagents were of the highest grade available.

### Cloning of *nape-1* and *nape-2* into the pET28a expression vector

*nape-1* and *nape-2* were amplified from wild type cDNA with primers containing engineered restriction sites. These fragments were T/A cloned into the pGEM-T Easy vector (Promega) and sequenced. The corresponding fragments were then cloned into the *E. coli* expression vector pET28a (Novagen) using the NdeI/XhoI sites for *nape-1* and the XhoI/EcoRI sites for *nape-2*.

OverExpress C41(DE3) chemically competent cells (Lucigen) were transformed with pET28a*(nape-1)* or pET28a*(nape-2)* constructs. Recombinant polyhistidine-tagged NAPE-1 and NAPE-2 were expressed in C41(DE3) positive clones by 1 mM isopropyl-*β*-D-thiogalactopyranoside (IPTG) induction at an A_600_ of 0.6 for 4 hours at 37°C. Induced cells were pelleted at 7000× g for 20 min at 4°C, and stored overnight at −20°C. For recombinant protein purification, frozen cells were thawed and incubated on ice with 50 mM Tris buffer (pH 8.0) containing 100 mM NaCl and 0.2 mM n-dodecyl-*β*-D-maltoside (DDM) for 30 min, followed by sonication (6×10 s pulse with 10 s chill on ice) and centrifugation at 13000× g for 30 min. Cell lysates were then incubated with Ni-NTA agarose beads (Qiagen) for 120 min at 4°C. Ni-NTA beads were pelleted at 1000× g for 1 min; the beads were pipetted into columns and washed three times with 50 mM NaH_2_PO_4_ buffer (pH 8.0) containing 300 mM NaCl and 20 mM imidazole. Recombinant NAPE-1 and NAPE-2 were eluted from the Ni-NTA beads with 50 mM NaH_2_PO_4_ buffer (pH 8.0) containing 300 mM NaCl and 250 mM imidazole. Purified proteins were analyzed by SDS-PAGE (NuPAGE 4–12% Bis Tris gel) and their concentrations were determined using the Pierce 660 nm protein assay kit (Thermo Fisher) and immediately used for activity measurements.

### Enzyme assays

The phospholipase-D activities of purified NAPE-1 and NAPE-2 were assayed in 200 µL Tris buffer (100 mM, pH 7.6) containing 0.5 mM DDM and 1 mM CaCl_2_ with *N*-arachidonoyl PE or *N*-palmitoyl PE as substrate [5]. Known amounts of the substrate were dried and re-suspended in the buffer mixture followed by addition of 1 µg of purified protein. The reactions were carried out for 4 hours at 20°C, and terminated by extraction twice with 400 µL hexane. Lipid extracts were dried down, derivatized with BSTFA and analyzed by GC-MS as described previously [17]. AEA-*d*4 and PEA-*d*4 were used as a standard.

### *C. elegans* maintenance and strains

*C. elegans* strains were maintained as previously described [18]. Bristol N2 (wild-type), *daf-2(e1368) III*, *fat-1(ok2323) IV*, *fat-4(ok958) IV* and *fat-3(wa22) IV* were obtained from the *Caenorhabditis elegans* Genetics Center (University of Minnesota, MN). *faah-1(tm5011) IV*, *nape-1(tm2860) IV* and *nape-2(tm6254) IV* were obtained from Dr Shohei Mitani at the National Bioresource Project at Tokyo Women's Medical University School of Medicine and each strain was backcrossed 5 times to wild type prior to analysis.

### Generation of transgenic lines

Translational C-terminal GFP or mCherry fusions were generated essentially as described by Hobert [19]. To generate the *pnape-1::nape-1::mCherry* construct, *nape-1* coding sequence was amplified with 2 kb of the promoter region and fused to an *mCherry::unc-54* 3′UTR fragment. For generation of *pnape-2::nape-2::gfp*, *nape-2* coding sequence and approximately 1.7 kb of the promoter region was fused to a *gfp::unc-54* 3′UTR fragment of pPD95.75 (gift from Andrew Fire). The resulting constructs were microinjected into the gonad of young adult worms and transgenic animals were identified in the next generation by their expression of the co-injection marker *unc-25::mrfp*. At least four independent transgenic lines were analyzed. A representative line was chosen for integration using trimethylpsoralen/UV mutagenesis. The resulting integrated lines, containing the transgenes *jluIs7(nape-1::mCherry unc-25::mrfp)* and *jluIs2(nape-2::GFP unc-25::mrfp)*, were outcrossed to wild type five times before analysis and are referred to as *nape-1(OE)* and *nape-2(OE)*, respectively. To visualize co-localization of *nape-1::mCherry* and *nape-2::gfp* without interference from the co-injection marker a non-integrated transgenic line was generated by co-injecting *pnape-1::nape-1::mCherry* and *pnape-2::nape-2::gfp* without the *unc-25::mrfp* marker (resulting line *jluEx71(nape-1::mCherry nape-2::gfp)*.

### Genetic crosses

A strain over-expressing both *nape-1* and *nape-2* was generated by crossing *jluIs7* with *jluIs2*. The *nape* single over-expressers were also crossed into the *faah-1(tm5011)*, *daf-2(e1368)*, *fat-1(ok2323)*, *fat-4(ok958)* and *fat-3(wa22)* mutant backgrounds by standard methods. A *daf-2(e1368); fat-4(ok958)* double mutant was also generated and crossed with *fat-4(ok958); jluIs7* and *fat-4(ok958); jluIs2* to generate *daf-2(e1368); fat-4(ok958); jluIs7* and *daf-2(e1368); fat-4(ok958); jluIs2*, respectively. Mutants containing *daf-2(e1368)* were verified by direct sequencing of the *daf-2* mutation, while *faah-1(tm5011)*, *nape-1(tm3860)*, *nape-2(tm6254)*, *fat-1(ok2323)* and *fat-4(ok958)* genotypes were confirmed by PCR. Double mutants with *fat-3(wa22)* were confirmed by phenotype.

### Confocal imaging

Fluorescence images were acquired at 40× magnification using an Olympus FluoView 1000 laser scanning confocal microscope such that fluorescence was within the limits of under- and over-exposure. Image quantification was carried out using Image J software. Briefly, individual Z-stack images were combined into one image and normalized for brightness and contrast. Fluorescence intensity measurements were determined by subtracting background fluorescence from the region of interest (ROI). The size of the ROI was kept the same for all samples within a replicate.

### Growth assays

Worms were conditioned at each temperature for a minimum of two generations prior to examining growth, except for studies in the *fat* mutant backgrounds, in which worms were maintained at 20°C and not conditioned at the assay temperature. Eggs from a one hour synchronous lay were collected and incubated at 15°C, 20°C or 25°C for a time period sufficient for the wild type controls to reach adulthood (2 days at 25°C, 3 days at 20°C and 5 days at 15°C). Animals were scored for their developmental stage after this time period. Analysis was performed using Prism 4 software (Graphpad Software, Inc.). The raw data for the growth experiments are presented in Table S1.

### Dauer assays

Dauer assays were performed as previously described [20], [21]. In brief, for dauer entry assays, eggs from a synchronous lay were transferred to assay plates and incubated at 25°C. After 3 days, the plates were scored for the presence of dauer and non-dauer larvae based on morphology. For the dauer recovery assays, eggs from a synchronous lay were transferred to assay plates and incubated at 25°C. After 3 days, dauers were selected by treatment with 1% sodium dodecyl sulfate (SDS) for 15 minutes, transferred to a fresh plate and incubated at 20°C for 24 h. Recovery was scored by counting dauer and non-dauer animals after treatment with 1% SDS.

### Lifespan analysis

Lifespan analysis was performed as previously described [17]. Worms were maintained at 20°C and eggs from a synchronous lay were transferred to the appropriate temperature. For *daf-2* lifespans at 25°C, all strains were raised at 20°C and shifted to 25°C as first day adults to avoid dauer arrest. On the first day of adulthood, animals were transferred to plates containing 2 µg/mL 5-fluorodeoxyuridine (FUdR), to inhibit progeny production, with the exception of lifespans carried out at 15°C where no FUdR was used. Each condition was assayed in duplicate with 50 worms per plate. Worms were transferred to new plates every two days and transferred to plates without FUdR at day 8. Survival analysis was performed using Prism software (Graphpad Software, Inc.); Kaplan-Meier survival curves were plotted for each lifespan assay and compared using the Log-rank test. Animals that either crawled off the plate, experienced internal hatching or exhibited vulval protrusion were treated as censored objects in the survival analysis.

### Mass culture of worms for GC-MS

Mass cultures were maintained at 20°C. Starved L1s from eggs prepared by sodium hypochlorite treatment were used to seed 10 cm NGM plates spotted with 2 mL concentrated *E. coli* OP50. Adult worms were harvested from plates seeded with 20000–25000 starved L1s. For growth at 15°C and 25°C starved L1s from 20°C cultures were shifted to the appropriate temperature and harvested as adults (2 days at 25°C and 5 days at 15°C). Harvested worms were washed 3 times in S-basal, snap frozen in liquid nitrogen and stored at −80°C. A 20 µL aliquot was used to determine the total protein in the sample, using the bicinchoninic acid assay (BCA Protein Assay Kit, Thermo Scientific) with modifications for use with nematodes [22], [23].

### Measurement of NAE levels by GC-MS

For NAE quantification, lipid extraction and solid phase fractionation was carried out as previously described [17]. GC-MS analysis was carried out as previously described [17] with the following alterations. Samples were analyzed in triplicate, corresponding to at least 333 mg worms per run. Following derivatization with BSTFA for 60 min, samples were dried down under nitrogen to a volume of 1 µL. GC-MS analysis was performed using an Agilent 7890A GC and 240-ion trap MS system operating in splitless mode with a VF-5 ms capillary column (30 m×0.25 mm i.d., 5% phenyl-95% methyl polysiloxine, 0.25 µm film thickness; Varian, Inc., Walnut Creek, CA). GC conditions: Injector 280°C. Initial column temperature was 150°C for 1 min and then ramped at 20°C per min to 300°C followed by a 5°C ramp to 325°C and then held for 10 min. MS conditions: Analytes were chemically ionized using acetonitrile vapor and data was collected using the MRM mode in Agilent MS Workstation software version 7.0.0. Precursor ions were isolated using an isolation window of 3. MS/MS fragmentation was performed in the ion trap using an excitation storage level of 164.0, excitation amplitude of 80, and non-resonant collision energy for all analytes. Palmitoleoyl ethanolamine (POEA), linoleoyl ethanolamine (LOEA) and eicosapentaenoyl ethanolamine (EPEA) were quantified with reference to the PEA-*d4* internal standard and expressed as the amount of NAE per mg of protein.

## Results

**Figure 1:**
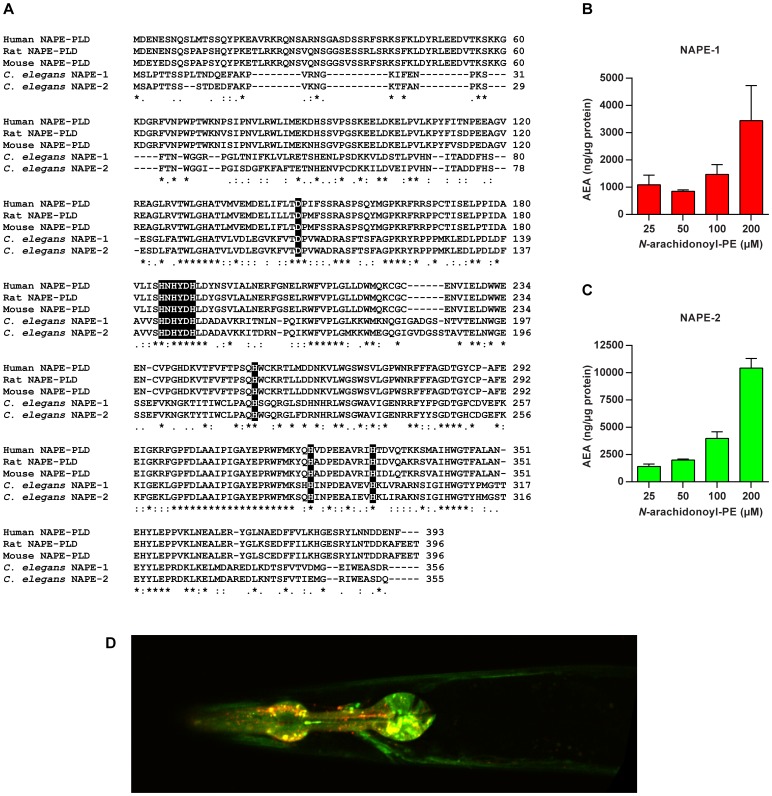
*C.elegans* NAPE-1 and NAPE-2 have NAPE-PLD activity and are expressed in overlapping and distinct tissues. (**A**) Alignment of *C. elegans* NAPE-1 and NAPE-2 amino acid sequences with human, mouse and rat NAPE-PLD. Both NAPE-1 and NAPE-2 contain all the key catalytic residues (highlighted in black boxes) characteristic of NAPE-PLD in mammals. Recombinant NAPE-1 (**B**) and NAPE-2 (**C**) are capable of generating arachidonoyl ethanolamine (AEA) from *N*-arachidonoyl phosphatidylethanolamine (N-arachidonoyl PE) *in vitro*. (**D**) Confocal microscopy image showing expression and co-localization of *pnape-1::nape-1::mCherry* and *pnape-2::nape-2::gfp* in the nervous system and pharynx.

There are two *C. elegans* genes with homology to the mammalian NAE biosynthetic enzyme NAPE-PLD, *nape-1* (Y37E11AR.4) and *nape-2* (Y37E11AR.3) that are adjacent to each other in the genome. *nape-1* and *nape-2* are predicted to express transcripts of 1348 bp and 3077 bp respectively, sharing 73% identity at the DNA sequence level, and producing similar sized protein products that have 84% identity and 92% similarity (Figure 1A). Both NAPE-1 and NAPE-2 retain all of the highly conserved residues in the NAPE-PLD signature sequence HX(E/H)XD(C/R/S/H)X_50–70_HX_15–30_(C/S/D)X_30–70_H [7], [24].

To confirm that the *C. elegans* NAPE-PLD orthologs are capable of synthesizing NAEs, we expressed recombinant His-tagged NAPE-1 and NAPE-2 in *E. coli* C41(DE3) cells from the pET28a expression vector and examined their ability to generate NAEs *in vitro*. Expression of the recombinant proteins was confirmed by SDS PAGE and the identities of purified NAPE-1 and NAPE-2 confirmed by LC/MS (Figure S1). Freshly purified NAPE-1 and NAPE-2 were capable of liberating arachidonoyl ethanolamine (AEA) from its precursor molecule *N*-arachidonoyl PE (Figure 1B and C), and no such AEA release was observed in controls lacking any protein (Figure S1). Similar results were obtained with another NAPE-PLD substrate, *N*-palmitoyl PE, which yielded palmitoyl ethanolamine (Figure S1). These data indicate that both *C. elegans* NAPE-1 and NAPE-2 are capable of generating NAEs and confirms that they are functional orthologs of NAPE-PLD.

To examine where *nape-1* and *nape-2* are expressed *in vivo*, we generated a transgenic strain that expressed each of the enzymes, under the control of their endogenous promoters, fused to either mCherry (*nape-1*) or GFP (*nape-2*) fluorescent tags. Both enzymes are expressed predominantly in the pharynx (Figure 1D), but *nape-2* is also highly expressed in the dorsal and ventral nerve cords, as well as being strongly expressed in the vulval muscles of hermaphrodites (Figure S2). *nape-1* is also expressed in these areas, but at very low levels, and is only seen in the cell bodies of the dorsal and ventral nerve cords (Figure S2).

**Figure 2:**
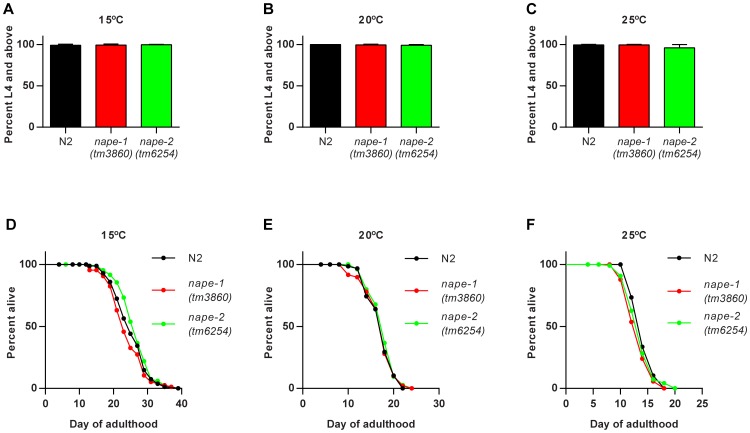
Deletion of *nape-1* or *nape-2* has no effect on growth or lifespan. There was no effect of the *nape-1(tm3860)* or *nape-2(tm6254)* deletions on growth at (**A**) 15°C, (**B**) 20°C or (**C**) 25°C compared with the wild type N2 strain. Data are presented as mean+s.d for 6 (15°C), 3(20°C) and 5 (25°C) biological replicates. (**D–F**) Lifespan was not significantly affected by *nape-1* or *nape-2* deletion at any temperature. Data shown are representative of three biological replicates. Summary data, statistical analysis and additional lifespan data are presented in Figure S3.

We had previously observed that over-expression of the NAE degrading enzyme *faah-1* resulted in slowed growth and increased lifespan [17]. We therefore predicted that loss of *nape-1* or *nape-2* might lead to a similar phenotype. The *nape-1* deletion mutant has a 483 bp in frame deletion that spans exons 2 and 3 and results in a predicted 214 aa peptide that lacks part of the NAPE-PLD signature sequence. The *nape-2* deletion mutant has a 476 bp deletion/32 bp insertion that removes exon 1 and part of exon 2. After backcrossing to wild type, neither the *nape-1* nor *nape-2* deletion had any effect on growth or lifespan at any temperature (Figure 2 and S3).

**Figure 3:**
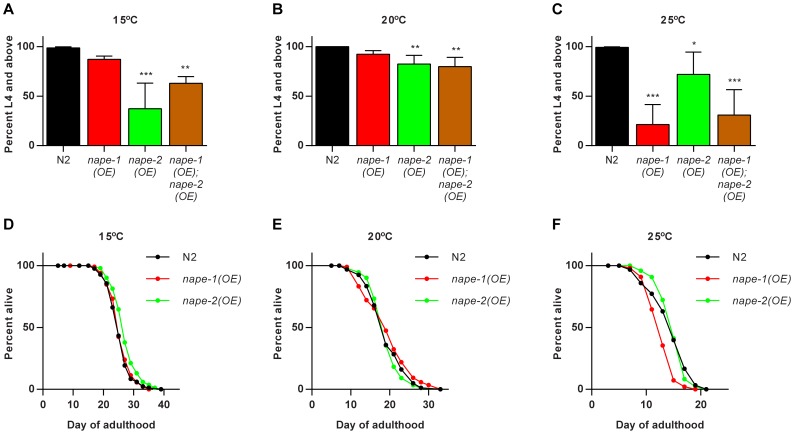
Over-expression of *nape-1* and *nape-2* affect development and lifespan in a temperature dependent manner. (**A**) At 15°C, *nape-2(OE)* worms showed significant growth delay that was characterized by L1 arrest, while the phenotype of *nape-1(OE)* worms was not different from controls and *nape-1(OE); nape-2(OE)* animals showed an intermediate phenotype (mean+sd, n = 9 except *nape-1(OE); nape-2(OE)* where n = 4, ANOVA p<0.0001, pairwise comparisons vs N2 **p<0.01, ***p<0.001). (**B**) At 20°C, *nape-2(OE)* and *nape-1(OE); nape-2(OE)* showed mild growth delay compared with N2 (n = 4, ANOVA p<0.01, pairwise comparison vs N2 **p<0.01). (**C**) At 25°C, *nape-1(OE)* worms showed the strongest growth delay, which was mirrored by the *nape-1(OE); nape-2(OE)* strain (n = 9 except *nape-1(OE); nape-2(OE)* where n = 4, ANOVA p<0.0001, pairwise comparisons vs N2 *p<0.05, ***p<0.001). (**D**) At 15°C, *nape-2(OE)* showed a small but significant increase in lifespan (p<0.01) while *nape-1(OE)* were not different compared with wild type. (**E**) At 20°C, there was no significant effect of *nape-1(OE)* or *nape-2(OE)* on lifespan. (**F**) At 25°C, *nape-1(OE)* showed a significant reduction in lifespan (p<0.0001), but there was no effect of *nape-2(OE)*. Data shown are representative of replicate experiments. Summary data, statistical analysis and additional lifespan data are presented in Figures S4, S5, S6.

Since there was no strong phenotype in either the *nape-1* or *nape-2* deletion mutants, we examined the effect of over-expression of each enzyme under the control of their respective native promoters. Given the level of homology between the two isoforms we hypothesized that over-expression of both *nape-1* and *nape-2* together would result in a stronger phenotype than either over-expresser alone. When grown at the standard temperature of 20°C, there was a subtle, low penetrance growth delay in the *nape-2(OE)* strain that was maintained, but not strengthened in the *nape-1(OE); nape-2(OE)* animals (Figure 3B). Interestingly, the growth phenotypes of the *nape* over-expressing strains diverged when grown at 15°C and 25°C. At 15°C, *nape-1(OE)* showed mild growth delay similar to that seen at 20°C, but in *nape-2(OE)* at 15°C there was a significant fraction of animals that arrested at the first larval stage (L1) of development, with only 37% of animals reaching larval stage 4 (L4) after 5 days, compared with 99% in wild type (Figure 3A). Interestingly, at this temperature, the phenotype of the *nape-1(OE); nape-2(OE)* animals more closely resembled the *nape-1(OE)* strain, suggesting that the effects of *nape-2(OE)* were mitigated by the presence of the *nape-1* transgene (Figure 3A). In contrast, at 25°C *nape-2(OE)* animals showed a mild growth defect, but in *nape-1(OE)* animals there was a pronounced growth delay with only 21% of animals reaching the L4 stage after 2 days, compared with 99% in wild type (Figure 3C). At this temperature, animals over-expressing both *nape-1* and *nape-2* showed a phenotype similar to that of *nape-1* alone (Figure 3C).

In terms of lifespan, there was no strong effect of *nape-2* over-expression at any temperature, but at 15°C lifespan was slightly increased in 3/5 trials (Figure 3D–F, Figures S4, S5, S6). At 15°C and 20°C, lifespan of *nape-1* over-expressers was not different from wild type, but at 25°C *nape-1(OE)* lifespan was significantly reduced compared with N2 in 4/6 trials (Figure 3D–F, Figures S4, S5, S6). The phenotype of worms over-expressing both *nape-1* and *nape-2* tracked with *nape-2* at 15°C and with *nape-1* at 25°C (Figures S4, S5, S6). Taken together, the growth and lifespan data suggest that despite strong sequence homology and shared ability to generate NAEs *in vitro*, NAPE-1 and NAPE-2 may function differently *in vivo* in response to temperature.

**Figure 4:**
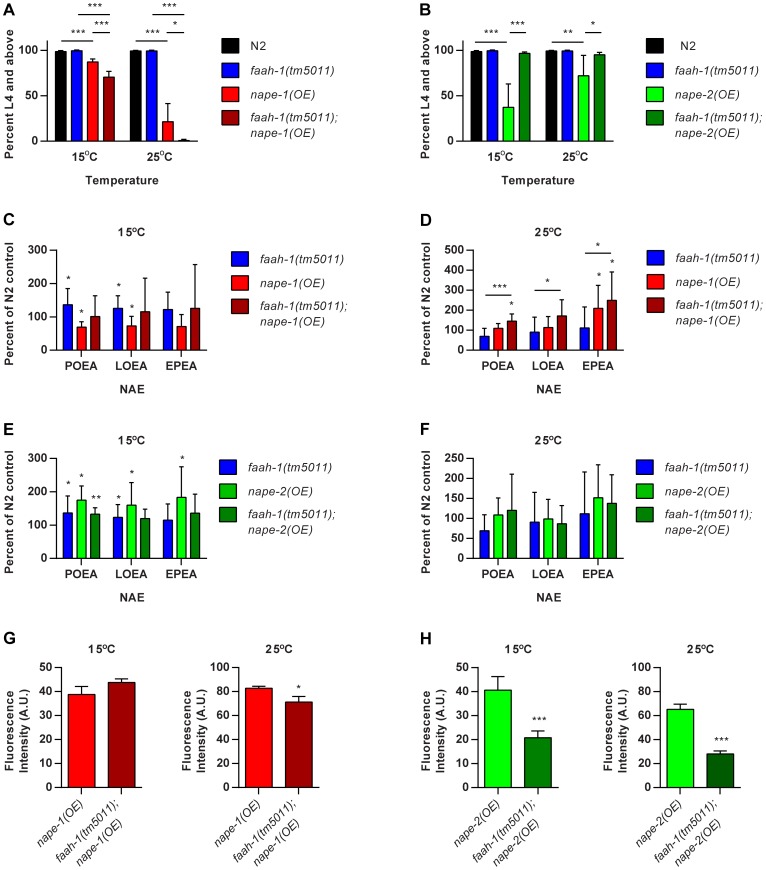
Loss of *faah-1* enhances *nape-1(OE)* phenotypes but suppresses *nape-2(OE)*. (**A**) *faah-1(tm5011)* enhances the growth delay of *nape-1* over-expressers at both 15°C and 25°C (mean+sd, N2 and *nape-1(OE)* n = 9 and others n = 5 replicates, ANOVA p<0.0001 at each temperature, *** p<0.001 and * p<0.05 for indicated pairwise comparisons). (**B**) *faah-1(tm5011)* suppresses the L1 arrest of *nape-2* over-expressers at both 15°C and 25°C (mean+sd, N2 and *nape-2(OE)* n = 9 and others n = 5 replicates, ANOVA p<0.0001 at each temperature, *** p<0.0001, ** p<0.01 and *p<0.05 for indicated pairwise comparisons). (**C**) NAE levels in *nape-1(OE)* and *faah-1(tm5011); nape-1(OE)* grown at 15°C were not significantly elevated (mean+sd, *faah-1(tm5011)* n = 12, *nape-1(OE)* and *faah-1(tm5011); nape-1(OE)* n = 7 replicates, one sample test for deviation from 100% - *p<0.05). (**D**) NAE levels in *nape-1(OE)* and *faah-1(tm5011); nape-1(OE)* grown at 25°C showed a significant linear trend towards increased levels (mean+sd, *faah-1(tm5011)* n = 9, *nape-1(OE)* n = 8 and *faah-1(tm5011); nape-1(OE)* n = 6 replicates, one sample test for deviation from 100% - *p<0.05). ***p<0.0001 and *p<0.05 for the linear trend are indicated above the solid lines. (**E**) At 15°C NAE levels in *nape-2(OE)*, but not *faah-1(tm5011); nape-2(OE)*, were elevated (mean+sd, *faah-1(tm5011)* n = 12, *nape-2(OE) and faah-1(tm5011); nape-2(OE)* n = 7 replicates, one sample test for deviation from 100% - *p<0.05). (**F**) NAE levels in *nape-2(OE)* and *faah-1(tm5011); nape-2(OE)* grown at 25°C were not different from N2 or from each other (mean+sd, *faah-1(tm5011)* n = 9, *nape-2(OE)* n = 6 and *faah-1(tm5011); nape-2(OE)* n = 5 replicates). (**G**) There was no change in NAPE-1::mCherry fluorescence intensity in the *faah-1(tm5011)* deletion background at 15°C and only a minor decrease at 25°C (*p<0.05). Data are from n = 10 worms for each strain and are representative of three independent replicates. (**H**) There was a significant reduction in NAPE-2::GFP expression in the *faah-1(tm5011)* deletion background at both 15°C and 25°C (***p<0.0001). Data are from n = 10 worms for each strain and are representative of three independent replicates at 15°C, and 2 independent replicates at 25°C.

We have previously shown that global over-expression of *faah-1* reduced NAE levels and that RNAi of *faah-1* increased NAE levels [17]. We therefore hypothesized that loss of *faah-1* activity would exacerbate the growth phenotypes conferred by *nape* over-expression by further elevating NAE levels. The *faah-1(tm5011)* mutant contains a 515 bp deletion that removes the second exon of the gene and is likely a null. Consistent with our hypothesis, we found that the *faah-1* deletion enhanced the growth delay of *nape-1* over-expressers at both 15°C and 25°C (Figure 4A). Surprisingly, loss of *faah-1* in the *nape-2(OE)* strain completely rescued the L1 arrest observed at both temperatures, implying that loss of *faah-1* mitigates the effects of *nape-2* over-expression (Figure 4B).

Given that NAPE-1 and NAPE-2 are capable of generating NAEs *in vitro* we hypothesized that NAE levels would be altered in the *nape* over-expressers. At 15°C, NAE levels in *nape-1(OE)* were not significantly elevated compared with control animals (Figure 4C). However, at 25°C EPEA in *nape-1(OE)* and POEA and EPEA in *faah-1(tm5011); nape-1(OE)* were higher than N2, and there was also a significant linear trend towards higher levels of each NAE levels across the strains (Figure 4D). These data are consistent with the appearance of stronger growth and lifespan phenotypes at 25°C in *nape-1* over-expressers and suggest that *nape-1(OE)* has a greater effect on NAE levels at 25°C compared to 15°C.

At 15°C NAE levels in *nape-2(OE)* were significantly higher than N2 but there was not a statistically significant increase at 25°C. This is consistent with stronger *nape-2(OE)* growth phenotypes at 15°C. NAE levels in *faah-1(tm5011); nape-2(OE)* did not show any statistically significant differences at either temperature, but there was a tendency for the higher levels in *nape-2(OE)* to be reduced by the presence of the *faah-1* deletion (Figure 4E & F). This is consistent with the observation that the *faah-1* deletion rescued the growth phenotype of *nape-2(OE)* animals (Figure 4B) and suggests that loss of *faah-1* reduces *nape-2* expression or activity. To provide a molecular basis for this effect, we examined the level of the fluorescently tagged NAPE-PLDs in the *faah-1* deletion background. There was no significant difference in *nape-1::mCherry* levels at 15°C and only a minor reduction at 25°C in *faah-1(tm5011); nape-1(OE)* compared with *nape-1(OE)*, suggesting that the increased levels of NAEs in the *faah-1(tm5011); nape-1(OE)* background was not due to an increase in *nape-1* protein (Figure 4G). In contrast, we found that there was a significant reduction in *nape-2::gfp* levels in the *faah-1* deletion background at both 15°C and 25°C (Figure 4H), suggesting that the reduction of NAEs in the *faah-1(tm5011); nape-2(OE)* strain could be due to reduced *nape-2* protein expression.

**Figure 5:**
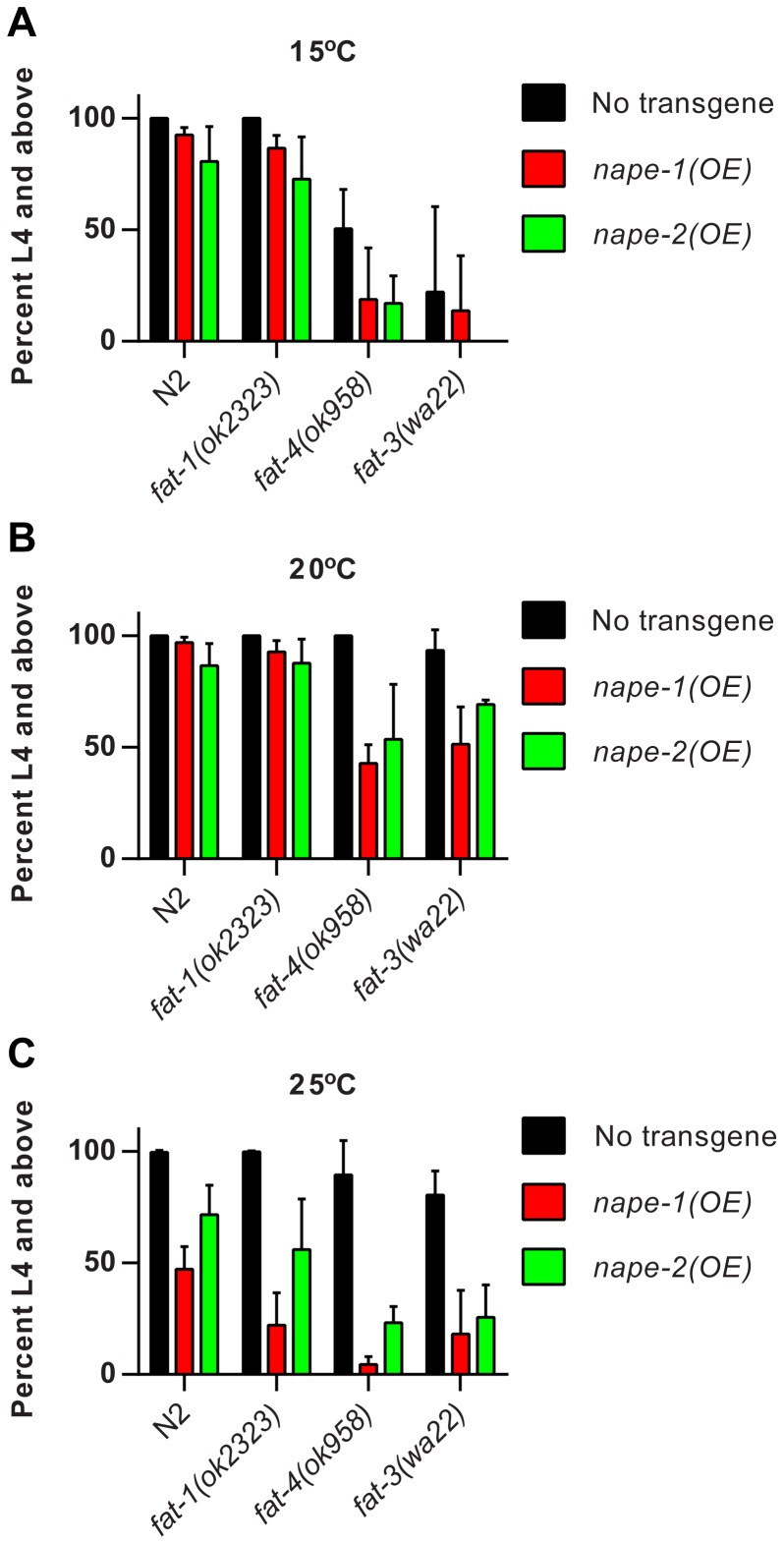
The absence of C20 fatty acids enhances the growth defects in *nape* over-expressers. (**A**) At 15°C, mutations in the fatty acid desaturases *fat-4* and *fat-3*, but not *fat-1*, confer developmental delay in all strains (No transgene - ANOVA p<0.001, pairwise comparisons N2 vs *fat-1* p = n.s, vs *fat-4* p<0.05 and vs *fat-3* p<0.001; *nape-1(OE)* – ANOVA p<0.0001, pairwise comparisons N2 vs *fat-1* p = n.s, vs *fat-4* p<0.001 and vs *fat-3* p<0.0001; *nape-2(OE)* – ANOVA p<0.0001, pairwise comparisons N2 vs *fat-1* p = n.s, vs *fat-4* p<0.0001 and vs *fat-3* p<0.0001). (**B**) At 20°C, mutations in the fatty acid desaturases have no effect in non-transgenic animals (ANOVA p = n.s.). However, growth of *nape-1(OE)* animals is influenced by *fat-4* and *fat-3* (ANOVA p<0.0001, pairwise comparisons N2 vs *fat-1* p = n.s, vs *fat-4* p<0.0001 and vs *fat-3* p<0.0001), whereas *nape-2(OE)* animals are adversely affected by *fat-4* only (ANOVA p<0.05, pairwise comparisons N2 vs *fat-1* p = n.s, vs *fat-4* p<0.05 and vs *fat-3* p = n.s.). (**C**) At 25°C, there is a mild effect of the *fat-3* mutation on growth of non-transgenic animals (ANOVA p<0.05, pairwise comparisons N2 vs *fat-1* p = n.s., vs *fat-4* p = n.s. and vs *fat-3* p<0.05). At this temperature, the growth defect of *nape-1(OE)* animals is further enhanced by all *fat* mutants (ANOVA p<0.0001; pairwise comparisons *nape-1(OE)* vs *fat-1* p<0.05, vs *fat-4* p<0.001 and vs *fat-3* p<0.05). Growth of *nape-2(OE)* animals was affected only by *fat-4* and *fat-3*, but not *fat-1* (ANOVA p<0.0001; pairwise comparisons *nape-2(OE)* vs *fat-1* p = n.s., vs *fat-4* p<0.001 and vs *fat-3* p<0.001). Data are presented as mean+sd for 4 replicates at 15°C and 20°C and 5 replicates at 25°C. The milder growth phenotype of the *nape-2(OE)* animals at 15°C in these experiments is due to the fact that these animals had not been conditioned at the lower temperature prior to growth studies.

The NAE measurements indicated a tendency toward elevated EPEA levels in the *nape-1* over-expressing strain at 25°C, where this strain showed the strongest growth delay. In our previous study we had observed that exogenous treatment of wild type animals with EPEA did not have any deleterious effect on growth and fertility [17], suggesting that other NAEs may be responsible for the growth delay. To examine growth in the absence of C20 NAEs such as EPEA, we crossed the *nape* over-expressing strains with mutants that are unable to synthesize the fatty acid precursors for these long-chain NAEs. *fat-1*, *fat-4* and *fat-3* encode fatty acid desaturases and mutations in these genes lead to specific fatty acid defects [25]. Loss of *fat-1* results in the absence of eicosapentaenoic acid, the precursor for EPEA, and a corresponding increase in arachidonic acid, the precursor for AEA. Mutation of *fat-4* leads to a failure to synthesize both arachidonic and eicosapentaenoic acid, but other C20 fatty acids are still present. Finally, loss of *fat-3* generates an animal devoid of C20 polyunsaturated fatty acids. Loss of either *fat-4* or *fat-3*, but not *fat-1*, resulted in growth delay in wild type and *nape* over-expressers at 15°C (Figure 5A). However, at 20°C, although there was no effect of either of the *fat* mutations on growth in non-transgenic animals, loss of *fat-4* or *fat-3* delayed growth in *nape-1(OE)*, and loss of *fat-4* significantly delayed growth in *nape-2(OE)* animals (Figure 5B). At 25°C, the delayed growth of *nape-1(OE)* animals was further exacerbated by each *fat* mutant, while only *fat-4* and *fat-3* further affected *nape-2(OE)* animals (Figure 5C). Taken together, these data suggest that the presence of C20 fatty acids, and by inference C20 NAEs, mitigates the deleterious effects of *nape* over-expression on growth and implies that it is NAEs other than EPEA that are causing the growth delay.

**Figure 6:**
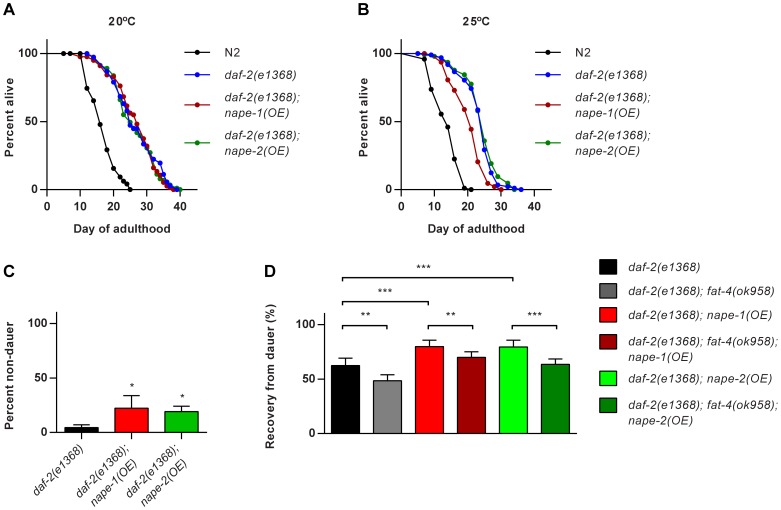
*nape* over-expression modulates lifespan and dauer formation in *daf-2* mutants. (**A**) *nape-1* or *nape-2* over-expression has no effect on lifespan of *daf-2(e1368)* mutants at 20°C. (**B**) *nape-1(OE)*, but not *nape-2(OE)*, suppresses *daf-2(e1368)* lifespan at 25°C (p<0.0001). (**C**) *nape-1(OE)* and *nape-2(OE)* have a small but significant suppressive effect on dauer formation in *daf-2(e1368)* at 25°C (mean+sd, n = 4 replicates). (**D**) *nape-1(OE)* and *nape-2(OE)* enhances dauer recovery in *daf-2(e1368)*. Loss of the fatty acid desaturase *fat-4*, which eliminates EPEA and AEA, leads to reduced dauer recovery in *daf-2(e1368)*, *daf-2(e1368); nape-1(OE)* and *daf-2(e1368); nape-2(OE)* (mean+sd, n = 9 trials from 3 biological replicates ANOVA p<0.0001, pairwise comparisons *p<0.05, **p<0.01, ***p<0.001).

In our previous study we had also observed that EPEA treatment suppressed dauer entry and reduced lifespan in *daf-2* insulin receptor mutants [17]. We therefore examined the effect of *nape* over-expression on dauer formation and lifespan in *daf-2* mutants. There was no effect of either *nape-1(OE)* or *nape-2(OE)* on *daf-2(e1368)* lifespan at 20°C, but at 25°C *nape-1(OE)* resulted in a significant reduction in *daf-2* lifespan (Figure 6A & B, Figures S5 & S7). There was also a small suppression of dauer formation in both the *nape* over-expressers at the restrictive temperature of 25°C (Figure 6C), as well as an increase in the number of animals that recovered from dauer after 24 h at the permissive temperature of 20°C (Figure 6D). These data suggest that an increase in the endogenous level of EPEA enhances dauer recovery. To test this further, we examined dauer recovery in a *daf-2*; *fat-4* mutant background and found a significant reduction in basal dauer recovery in the *daf-2; fat-4* double mutant compared with *daf-2* alone (Figure 6D). Loss of *fat-4* in the *daf-2; nape-1(OE)* and *daf-2; nape-2(OE)* backgrounds also reduced dauer recovery (Figure 6D), supporting the idea that endogenous EPEA levels influence dauer recovery.

## Discussion

We have previously determined that *C. elegans* has a functional NAE system, through the identification of multiple NAE molecules and the observation that the activity of the NAE degradation enzyme, FAAH, was conserved [17]. In this study, we have focused on characterizing the worm orthologs of the NAE biosynthetic enzyme NAPE-PLD.

In mammals, and other species, there is a single NAPE-PLD that generates NAEs from the phospholipid *N*-acyl phosphatidyl ethanolamine (NAPE). Comparative genomics had previously identified Y37E11AR.4 (*nape-1*) as a *C. elegans* ortholog of NAPE-PLD [9] and we had identified another candidate, (Y37E11AR.3, *nape-2*). Given their sequence similarity, and their adjacent location in the genome, it is highly likely that *nape-1* and *nape-2* arose from a gene duplication event, a phenomenon that is common in *C. elegans* [26], [27]. Here we show that the recombinant proteins, expressed and purified from bacteria, retain their NAPE-PLD activity and when over-expressed in *C. elegans nape-1* and *nape-2* modulate NAE levels, consistent with their proposed function. NAPE-2 appeared to be more effective in the *in vitro* assays, but whether this is due to differences in the protein preparations or a true reflection of substrate preference remains to be determined.

Translational fusions of *nape-1* and *nape-2*, driven by their respective endogenous promoters, indicated that expression was primarily in the pharynx, which is consistent with the expression of a *nape-1* transcriptional fusion and the expression of *faah-1* [17]. However, *nape-2* appeared to be more broadly expressed, with fluorescence also observed in the nerve ring, the dorsal and ventral nerve cords, and the reproductive system. The overlap in their expression pattern was restricted to small distinct areas within the pharynx suggesting that *nape-1* and *nape-2* may have undergone functional divergence, which is common in duplicated genes in *C. elegans* [26]. However, the fact that each enzyme was able to generate both a short chain, saturated NAE (PEA) and a long-chain, polyunsaturated NAE (AEA) *in vitro*, suggests that spatial and temporal differences in expression pattern or substrate availability may drive the difference in function more than differences in substrate specificity.

In our previous work, reduced NAE levels, arising from over-expression of *faah-1*, were associated with delayed growth [17], but in the current study, single deletion mutants of *nape-1* and *nape-2* did not affect growth at any temperature. It is possible that a deletion of both *nape-1* and *nape-2* would have a stronger phenotype than either single mutant, but we were unable to generate such a mutant because of the close proximity of the two genes in the genome. However, it is also possible that there are alternate pathways generating NAEs in *C. elegans* that are independent of NAPE-PLD, as has been observed in mammals [7], [28]–[32], which could minimize the effect of *nape* deletion.

Given that neither *nape-1* nor *nape-2* deletion conferred an obvious phenotype, we focused on phenotypes associated with *nape-1* or *nape-2* over-expression. Surprisingly, *nape-1* over-expression conferred a generalized delay in development, while *nape-2* over-expression resulted in a partially penetrant L1 arrest phenotype. Perhaps more surprising was the observation that growth and lifespan phenotypes varied with temperature, with *nape-1* over-expressers more severely affected at 25°C and *nape-2* over-expressers more adversely affected at 15°C. There did not appear to be any major changes in transgene expression at each temperature that could explain the phenotypic differences (Figure S8). In many other systems NAPE-PLD and FAAH appear to have broad specificities suggesting that the exact composition of the NAE profile is a consequence of other factors such as substrate availability and/or composition [1], [33].

The NAE system is thought to be an on-demand system, in that the activity of a given NAE is determined by the balance between its synthesis and degradation [1]. Based on this, we reasoned that loss of function of the degrading enzyme encoded by *faah-1* would enhance the phenotypes associated with NAPE-PLD over-expression. We found this to be the case for the *faah-1(tm5011); nape-1(OE)* strain, but in contrast, we found that loss of *faah-1* suppressed the L1 arrest phenotype of the *nape-2(OE)* strain. Further characterization of this strain indicated that there was a reduction in *nape-2::gfp* expression from the transgene in the presence of the *faah-1* deletion. One explanation for this phenomenon is that there is a negative feedback loop in which high NAE levels arising from *faah-1* deletion down-regulate *nape-2* expression, to maintain NAE levels within a physiological range. Such a mechanism has been proposed both in mice and *Arabidopsis* [34], [35]. Alternatively, there may be as yet unidentified NAEs that are produced independently of NAPE-2, and that are metabolized by FAAH-1, that act to suppress *nape-2* expression. Interestingly, such a feedback loop does not appear to operate with *nape-1*, since loss of *faah-1* exacerbated the *nape-1(OE)* growth phenotype. One possibility is that *nape-1* activity is coupled to one of the other *faah* orthologs that are found in the *C. elegans* genome that have yet to be characterized. Taken together, these data point toward differences in the regulation of the two NAPE-PLD homologs, and further support the functional divergence between the two.

The magnitude of the increase in NAEs in the *nape* over-expressing strains was relatively small. This could be related to the fact that NAEs are likely to signal over short distances, and thus small changes in the local NAE environment may have a large phenotypic effect and not be reflected in systemic or global NAE levels. Moreover, measurement of NAEs in each over-expression strain did not identify specific changes that could explain differences in the *nape-1(OE)* and *nape-2(OE)* phenotypes. However, a disadvantage of the GC-MS method we use to measure NAE is that we only detect what we look for and thus there may be other, as yet undescribed NAE species that are altered in response to *nape* over-expression which could potentially be detected with an unbiased analytical approach [35], [36]. Another possibility, that we were not able to test in the current study, is that the phospholipid substrate for NAPE-PLD, NAPE, itself has a specific biological function. Thus, over-expressing the *nape* enzymes could result in changes in NAPE availability in specific tissues which affect developmental and aging phenotypes. In this respect, a specific biological function for NAPE has been proposed in other species [35], [37].

To date we have not identified a biological role for the C16 and C18 NAEs found in worms. However, the exacerbation of the growth defects in *nape* over-expressers lacking C20 fatty acids and NAEs suggests that one or more of the shorter chain NAEs could be responsible for the deleterious effects on growth. In contrast, we had previously observed that EPEA suppressed entry into the dauer larval stage, and reduced adult lifespan [17]. The observation that *nape-1(OE)*, but not *nape-2(OE)*, suppressed lifespan in both wild type, as well as *daf-2* mutants is consistent with the observed elevation in EPEA levels in this strain at 25°C. A new finding in the current study was that recovery from the dauer stage was augmented in *daf-2* mutants carrying the *nape* transgenes. This phenotype appears to be, at least in part, related to EPEA since we were able to show that loss of EPEA, via inactivation of the fatty acid desaturase *fat-4*, resulted in reduced recovery from the dauer larval stage in both the *daf-2* single mutant and in *daf-2* animals carrying either *nape-1(OE)* or *nape-2(OE)* transgenes. Taken together these data suggest that EPEA may have a role in signaling resumption of reproductive growth following arrest in the dauer larval stage.

In summary, we have shown that the *C. elegans* orthologs of NAPE-PLD, NAPE-1 and NAPE-2, are able to synthesize NAEs *in vitro*, in a manner that is consistent with a conservation of biochemical function, and further reinforces the nematode as a genetic model system for studying NAE physiology. While the response to environmental temperature in poikilotherms such as *C. elegans* has conventionally been viewed as a passive thermodynamic response, recent studies suggest that different genetic programs modulate the response to high and low temperatures, particularly with respect to aging [38], [39]. The differing effects of *nape-1* and *nape-2* over-expression in response to temperature could suggest a role for NAE signaling in these genetically encoded thermal response programs.

## Supporting Information

Figure S1 **Recombinant NAPE-1 and NAPE-2 generate** ***N*** **-acylethanolamines from** ***N*** **-acyl phosphatidylethanolamine substrates** ***in vitro*** **.** His-tagged NAPE-1 and NAPE-2 were expressed in *E. coli* C41(DE3) cells and the identity of the purified protein was confirmed by LC-MS. Two samples were analyzed for each protein and peptide coverage for a representative sample is shown for NAPE-1 (**A**) and NAPE-2 (**B**). (**C**) NAPE-1 and NAPE-2 generate AEA from *N*-arachidonoyl PE, and no AEA is detected from control reactions that lack protein. Both NAPE-1 (**D**) and NAPE-2 (**E**) also liberated PEA *in vitro* from *N*-palmitoyl PE substrate.
(DOCX)

Figure S2 ***nape-1::mCherry*** **and** ***nape-2::gfp*** **expression in other tissues.** Confocal microscopy images showing *pnape-1::nape-1::mCherry* (**A&D**) and *pnape-2::nape-2::gfp* (**B&E**) and co-localization (**C&F**). **A–C** show representative confocal images indicating that *nape-2* but not *nape-1* is expressed in the dorsal nerve cord. **D–F** show representative confocal images indicate that *nape-2* is expressed in the vulva.
(DOCX)

Figure S3 **Summary of lifespan experiments with** ***nape-1*** **and** ***nape-2*** **deletion strains.**
(DOCX)

Figure S4 **Summary of lifespan experiments with** ***nape*** **over-expressing strains at 15°C.**
(DOCX)

Figure S5 **Summary of lifespan experiments with** ***nape*** **over-expressing strains at 20°C.**
(DOCX)

Figure S6 **Summary of lifespan experiments with** ***nape*** **over-expressing strains at 25°C.**
(DOCX)

Figure S7 **Summary of lifespan experiments with** ***nape*** **over-expressers in the** ***daf-2*** **background at 25°C.**
(DOCX)

Figure S8 ***nape-1::mCherry*** **and** ***nape-2::gfp*** **expression is not affected by changes in growth temperature.** Confocal images of *nape-1(OE)* and *nape-2(OE)* worms grown at (A) 15°C and (B) 25°C. The dashed lines in the *nape-1(OE)* images indicate the *unc-25::mrfp* co-injection marker.
(DOCX)

Table S1 **Raw growth data for** **Figures 2A–C** **,** **3A–C** **,** **4A&B** **and** **5A–C** **.**
(DOCX)
