# Supplementary material for: Characterization of N-Acyl Phosphatidylethanolamine-Specific Phospholipase-D Isoforms in the Nematode Caenorhabditis elegans
Source: PLoS One. 2014 Nov 25;9(11):e113007. doi: 10.1371/journal.pone.0113007 (PMC4244089; doi:10.1371/journal.pone.0113007)
Supplement: Figure S1 — Recombinant NAPE-1 and NAPE-2 generate N -acylethanolamines from N -acyl phosphatidylethanolamine substrates in vitro . His-tagged NAPE-1 and NAPE-2 were expressed in E. coli C41(DE3) cells and the identity of the purified protein was confirmed by LC-MS. Two samples were analyzed for each protein and peptide coverage for a representative sample is shown for NAPE-1 (A) and NAPE-2 (B). (C) NAPE-1 and NAPE-2 generate AEA from N-arachidonoyl PE, and no AEA is detected from control reactions that lack protein. Both NAPE-1 (D) and NAPE-2 (E) also liberated PEA in vitro from N-palmitoyl PE substrate. (DOCX) [file pone.0113007.s001.docx]

**Figure S1:** **Recombinant NAPE-1 and NAPE-2 generate *N-*acyl ethanolamines from *N*-acyl phosphatidylethanolamine substrates *in vitro*.** His-tagged NAPE-1 and NAPE-2 were expressed in *E. coli* C41(DE3) cells and the identity of the purified protein was confirmed by LC-MS. Two samples were analyzed for each protein and peptide coverage for a representative sample is shown for NAPE-1 **(A)** and NAPE-2 **(B)**. **(C)** NAPE-1 and NAPE-2 generate AEA from *N*-arachidonoyl PE, and no AEA is detected from control reactions that lack protein. Both NAPE-1 **(D)** and NAPE-2 **(E)** also liberated PEA *in vitro* from *N*-palmitoyl PE substrate.

**A**


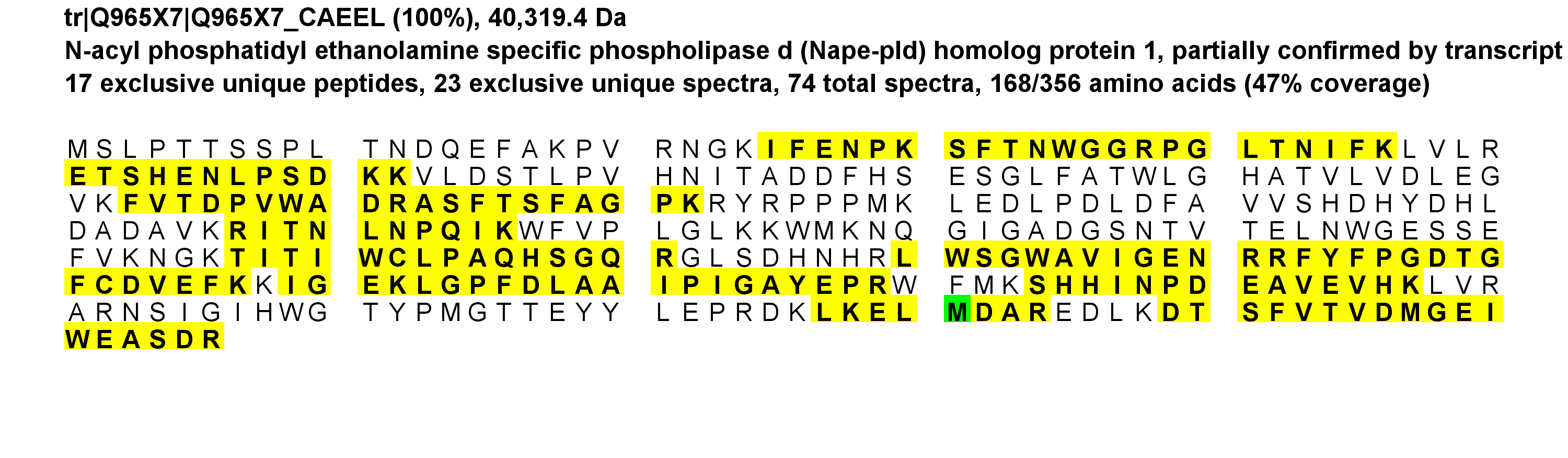


**B**


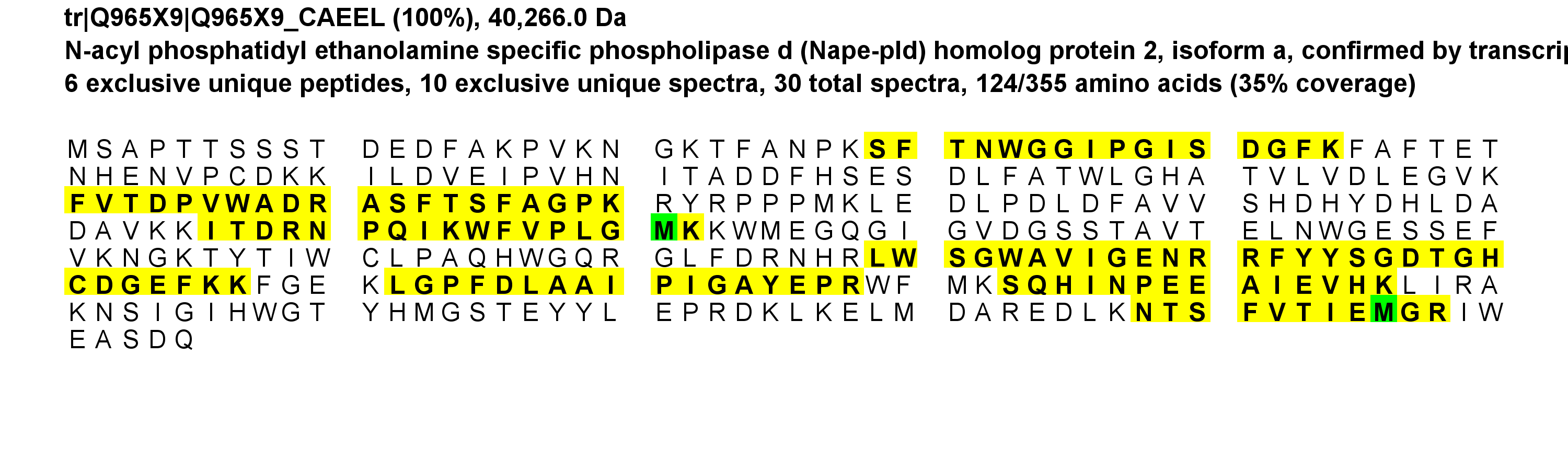


**C D E**
