# Supplementary material for: Characterization of N-Acyl Phosphatidylethanolamine-Specific Phospholipase-D Isoforms in the Nematode Caenorhabditis elegans
Source: PLoS One. 2014 Nov 25;9(11):e113007. doi: 10.1371/journal.pone.0113007 (PMC4244089; doi:10.1371/journal.pone.0113007)

**Figure S2:** ***nape-1::mCherry* and *nape-2::gfp* expression in other tissues.** Confocal microscopy images showing *pnape-1::nape-1::mCherry* **(A&D)** and *pnape-2::nape-2::gfp* **(B&E)** and co-localization **(C&F)**. **A-C** show representative confocal images indicating that *nape-2* but not *nape-1* is expressed in the dorsal nerve cord. **D-F** show representative confocal images indicate that *nape-*2 is expressed in the vulva.

**A B C**


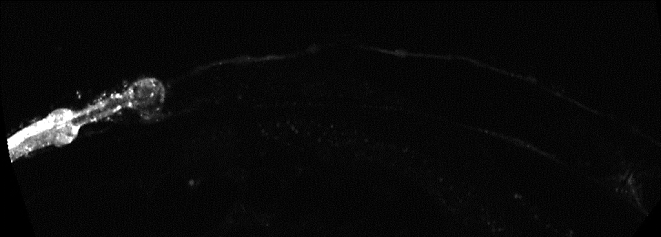

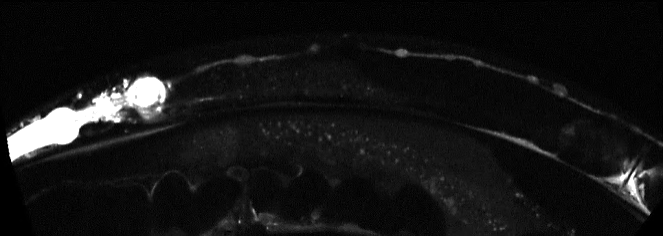

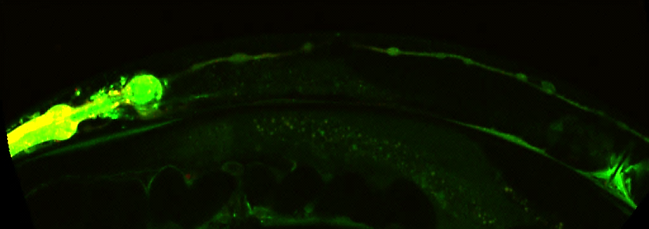


**D E F**


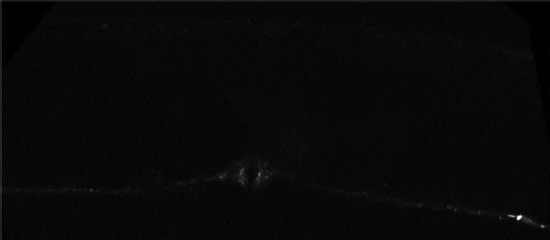

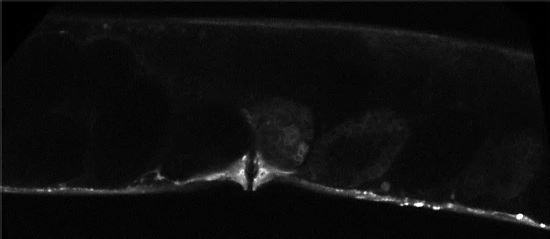

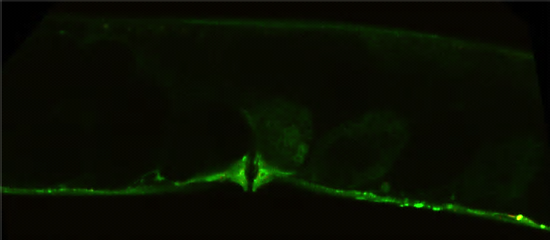

Supplement: Figure S2 — nape-1::mCherry and nape-2::gfp expression in other tissues. Confocal microscopy images showing pnape-1::nape-1::mCherry (A&D) and pnape-2::nape-2::gfp (B&E) and co-localization (C&F). A–C show representative confocal images indicating that nape-2 but not nape-1 is expressed in the dorsal nerve cord. D–F show representative confocal images indicate that nape-2 is expressed in the vulva. (DOCX) [file pone.0113007.s002.docx]
