# Supplementary material for: Characterization of N-Acyl Phosphatidylethanolamine-Specific Phospholipase-D Isoforms in the Nematode Caenorhabditis elegans
Source: PLoS One. 2014 Nov 25;9(11):e113007. doi: 10.1371/journal.pone.0113007 (PMC4244089; doi:10.1371/journal.pone.0113007)
Supplement: Figure S3 — Summary of lifespan experiments with nape-1 and nape-2 deletion strains. (DOCX) [file pone.0113007.s003.docx]

**Figure S3: Summary of lifespan experiments with *nape-1* and *nape-2* deletion strains.**

**(A)** 15°C

| **Trial** | **Genotype** | **Median survival** | **Deaths**  **(censored)** | **P value vs N2** | **P-value *nape-1 vs nape-2*** |
| --- | --- | --- | --- | --- | --- |
| ***Trial 1*** | N2 | 25 | 58 (42) | - | - |
|  | *nape-1(tm3860)* | 23 | 59 (45) | ns | - |
|  | *nape-2(tm6254)* | 23 | 67 (42) | <0.05 | ns |
| ***Trial 2**** | N2 | 25 | 82 (20) | - | - |
|  | *nape-1(tm3860)* | 23 | 80 (19) | ns | - |
|  | *nape-2(tm6254)* | 27 | 82 (28) | ns | <0.05 |
| ***Trial 3*** | N2 | 28 | 90 (18) | - | - |
|  | *nape-1(tm3860)* | 26 | 65 (45) | <0.05 | - |
|  | *nape-2(tm6254)* | 28 | 69 (34) | ns | ns |

* Data shown in Figure 2A

**(B)** 20°C

| **Trial** | **Genotype** | **Median survival** | **Deaths**  **(censored)** | **P value vs N2** | **P-value *nape-1 vs nape-2*** |
| --- | --- | --- | --- | --- | --- |
| ***Trial 1**** | N2 | 18 | 58 (43) | - | - |
|  | *nape-1(tm3860)* | 18 | 51 (50) | ns | - |
|  | *nape-2(tm6254)* | 18 | 74 (31) | ns | ns |
| ***Trial 2*** | N2 | 17 | 89 (13) | - | - |
|  | *nape-1(tm3860)* | 17 | 82 (24) | ns | - |
|  | *nape-2(tm6254)* | 19 | 95 (9) | <0.005 | ns |
| ***Trial 3*** | N2 | 20 | 83 (20) | - | - |
|  | *nape-1(tm3860)* | 18 | 63 (36) | <0.0001 | - |
|  | *nape-2(tm6254)* | 18 | 76 (22) | ns | <0.005 |

* Data shown in Figure 2B

**(C)** 25°C

| **Trial** | **Genotype** | **Median survival** | **Deaths**  **(censored)** | **P value vs N2** | **P-value *nape-1 vs nape-2*** |
| --- | --- | --- | --- | --- | --- |
| ***Trial 1*** | N2 | 14 | 95 (6) | - | - |
|  | *nape-1(tm3860)* | 14 | 73 (14) | ns | - |
|  | *nape-2(tm6254)* | 14 | 97 (1) | ns | ns |
| ***Trial 2**** | N2 | 14 | 78 (25) | - | - |
|  | *nape-1(tm3860)* | 14 | 88 (16) | <0.05 | - |
|  | *nape-2(tm6254)* | 14 | 96 (7) | ns | ns |
| ***Trial 3*** | N2 | 13 | 81 (17) | - | - |
|  | *nape-1(tm3860)* | 13 | 78 (12) | ns | - |
|  | *nape-2(tm6254)* | 15 | 77 (14) | ns | ns |

* Data shown in Figure 2C
