# Supplementary material for: Characterization of N-Acyl Phosphatidylethanolamine-Specific Phospholipase-D Isoforms in the Nematode Caenorhabditis elegans
Source: PLoS One. 2014 Nov 25;9(11):e113007. doi: 10.1371/journal.pone.0113007 (PMC4244089; doi:10.1371/journal.pone.0113007)
Supplement: Figure S4 — Summary of lifespan experiments with nape over-expressing strains at 15°C. (DOCX) [file pone.0113007.s004.docx]

**Figure S4: Summary of lifespan experiments with *nape* over-expressing strains at 15°C.**

| **Trial** | **Genotype** | **Median survival** | **Deaths**  **(censored)** | **P value vs N2** |
| --- | --- | --- | --- | --- |
| ***Trial 1*** | N2 | 25 | 70 (33) | - |
|  | *jluIs7 (nape-1::mCherry unc-25::mrfp)* | 27 | 87 (20) | <0.05 |
|  | *jluIs2 (nape-2::gfp unc-25::mrfp)* | 27 | 77 (21) | <0.001 |
|  | *jluIs7 (nape-1::mCherry unc-25::mrfp); jluIs2 (nape-2::gfp unc-25::mrfp)* | 27 | 40(14) | ns |
| ***Trial 2**** | N2 | 25 | 83 (26) | - |
|  | *jluIs7 (nape-1::mCherry unc-25::mrfp)* | 25 | 93 (17) | ns |
|  | *jluIs2 (nape-2::gfp unc-25::mrfp)* | 27 | 89 (15) | <0.005 |
|  | *jluIs7 (nape-1::mCherry unc-25::mrfp); jluIs2 (nape-2::gfp unc-25::mrfp)* | 27 | 81 (23) | <0.001 |
| ***Trial 3*** | N2 | 27 | 89 (9) |  |
|  | *jluIs7 (nape-1::mCherry unc-25::mrfp)* | 27 | 96 (8) | ns |
|  | *jluIs2 (nape-2::gfp unc-25::mrfp)* | 27 | 64 (45) | Ns |
| ***Trial 4*** | N2 | 27 | 89 (13) |  |
|  | *jluIs7 (nape-1::mCherry unc-25::mrfp)* | 29 | 81 (23) | <0.001 |
|  | *jluIs2 (nape-2::gfp unc-25::mrfp)* | 29 | 83 (34) | <0.001 |
| ***Trial 5*** | N2 | 29 | 86 (18) |  |
|  | *jluIs7 (nape-1::mCherry unc-25::mrfp)* | 29 | 75 (27) | ns |
|  | *jluIs2 (nape-2::gfp unc-25::mrfp)* | 27 | 67 (33) | ns |

* Data shown in Figure 3D
