# Supplementary material for: Characterization of N-Acyl Phosphatidylethanolamine-Specific Phospholipase-D Isoforms in the Nematode Caenorhabditis elegans
Source: PLoS One. 2014 Nov 25;9(11):e113007. doi: 10.1371/journal.pone.0113007 (PMC4244089; doi:10.1371/journal.pone.0113007)
Supplement: Figure S5 — Summary of lifespan experiments with nape over-expressing strains at 20°C. (DOCX) [file pone.0113007.s005.docx]

**Figure S5: Summary of lifespan experiments with *nape* over-expressing strains at 20°C.**

| **Trial** | **Genotype** | **Median survival** | **Deaths**  **(censored)** | **P value vs N2** | **P value vs *daf-2*** |
| --- | --- | --- | --- | --- | --- |
| ***Trial 1**** | N2 | 19 | 83 (18) | **-** | - |
|  | *jluIs7 (nape-1::mCherry unc-25::mrfp)* | 19 | 89 (14) | ns | - |
|  | *jluIs2 (nape-2::gfp unc-25::mrfp)* | 19 | 88 (15) | ns | - |
|  | *jluIs7 (nape-1::mCherry unc-25::mrfp); jluIs2 (nape-2::gfp unc-25::mrfp)* | 19 | 86 (19) | ns | - |
| ***Trial 2*** | N2 | 18 | 93 (12) | - | - |
|  | *jluIs7 (nape-1::mCherry unc-25::mrfp)* | 18 | 80 (21) | ns | - |
|  | *jluIs2 (nape-2::gfp unc-25::mrfp)* | 18 | 83 (20) | ns | - |
| ***Trial 3†*** | N2 | 16 | 97 (8) | - | - |
|  | *jluIs7 (nape-1::mCherry unc-25::mrfp)* | 16 | 98 (15) | ns | - |
|  | *jluIs2 (nape-2::gfp unc-25::mrfp)* | 18 | 95 (11) | <0.05 | - |
|  | *daf-2(e1368)* | 25 | 37 (64) | < 0.0001 | - |
|  | *daf-2(e1368); jluIs7 (nape-1::mCherry unc-25::mrfp)* | 27 | 75 (21) | < 0.0001 | ns |
|  | *daf-2(e1368); jluIs2 (nape-2::gfp unc-25::mrfp*) | 25 | 90 (7) | < 0.0001 | ns |
| ***Trial 4*** | N2 | 19 | 45 (6) | - | - |
|  | *jluIs7 (nape-1::mCherry unc-25::mrfp)* | 19 | 70 (26) | ns | - |
|  | *jluIs2 (nape-2::gfp unc-25::mrfp)* | 19 | 90 (14) | ns | - |
|  | *daf-2(e1368)* | 26 | 89 (14) | < 0.0001 | - |
|  | *daf-2(e1368); jluIs7 (nape-1::mCherry unc-25::mrfp)* | 30 | 70 (27) | < 0.0001 | <0.0001 |
|  | *daf-2(e1368); jluIs2 (nape-2::gfp unc-25::mrfp*) | 26 | 77 (23) | < 0.0001 | ns |

* Data shown in Figure 3E
† Data shown in Figure 6A
