# Supplementary material for: Characterization of N-Acyl Phosphatidylethanolamine-Specific Phospholipase-D Isoforms in the Nematode Caenorhabditis elegans
Source: PLoS One. 2014 Nov 25;9(11):e113007. doi: 10.1371/journal.pone.0113007 (PMC4244089; doi:10.1371/journal.pone.0113007)
Supplement: Figure S6 — Summary of lifespan experiments with nape over-expressing strains at 25°C. (DOCX) [file pone.0113007.s006.docx]

**Figure S6: Summary of lifespan experiments with *nape* over-expressing strains at 25°C.**

 **

| **Trial** | **Genotype** | **Median survival** | **Deaths**  **(censored)** | **P value vs N2** |  |
| --- | --- | --- | --- | --- | --- |
| ***Trial 1*** | N2 | 16 | 101 (3) | - |  |
|  | *jluIs7 (nape-1::mCherry unc-25::mrfp)* | 12 | 74 (41) | <0.0001 |  |
|  | *jluIs2 (nape-2::gfp unc-25::mrfp)* | 14 | 98 (4) | ns |  |
| ***Trial 2*** | N2 | 14 | 99 (5) | - |  |
|  | *jluIs7 (nape-1::mCherry unc-25::mrfp)* | 12 | 98 (4) | ns |  |
|  | *jluIs2 (nape-2::gfp unc-25::mrfp)* | 16 | 52 (50) | < 0.0001 |  |
|  | *jluIs7 (nape-1::mCherry unc-25::mrfp); jluIs2 (nape-2::gfp unc-25::mrfp)* | 14 | 91 (8) | ns |  |
| ***Trial 3*** | N2 | 15 | 90 (15) | - |  |
|  | *jluIs7 (nape-1::mCherry unc-25::mrfp)* | 12 | 93 (12) | ns |  |
|  | *jluIs2 (nape-2::gfp unc-25::mrfp)* | 15 | 95 (10) | <0.05 |  |
|  | *jluIs7 (nape-1::mCherry unc-25::mrfp); jluIs2 (nape-2::gfp unc-25::mrfp)* | 12 | 98 (6) | <0.01 |  |
| ***Trial 4*** | N2 | 15 | 89 (14) | - |  |
|  | *jluIs7 (nape-1::mCherry unc-25::mrfp)* | 13 | 98 (10) | <0.0001 |  |
|  | *jluIs2 (nape-2::gfp unc-25::mrfp)* | 15 | 48 (3) | ns |  |
| ***Trial 5**** | N2 | 15 | 91 (8) | - |  |
|  | *jluIs7 (nape-1::mCherry unc-25::mrfp)* | 13 | 97 (7) | <0.0001 |  |
|  | *jluIs2 (nape-2::gfp unc-25::mrfp)* | 15 | 97 (4) | ns |  |
| ***Trial 6*** | N2 | 14 | 91 (8) | - |  |
|  | *jluIs7 (nape-1::mCherry unc-25::mrfp)* | 12 | 97 (7) | <0.0001 |  |
|  | *jluIs2 (nape-2::gfp unc-25::mrfp)* | 14 | 97 (4) | ns |  |

* Data shown in Figure 3F
