# Supplementary material for: Characterization of N-Acyl Phosphatidylethanolamine-Specific Phospholipase-D Isoforms in the Nematode Caenorhabditis elegans
Source: PLoS One. 2014 Nov 25;9(11):e113007. doi: 10.1371/journal.pone.0113007 (PMC4244089; doi:10.1371/journal.pone.0113007)
Supplement: Figure S7 — Summary of lifespan experiments with nape over-expressers in the daf-2 background at 25°C. (DOCX) [file pone.0113007.s007.docx]

**Figure S7: Summary of lifespan experiments with *nap*e over-expressers in the *daf-2* background at 25°C.**

| **Trial** | **Genotype** | **Median survival** | **Deaths**  **(censored)** | **P value vs N2** | **P value vs**  ***daf-2*** |
| --- | --- | --- | --- | --- | --- |
| ***Trial 1*** | N2 | 14 | 101 (2) | - | - |
|  | *jluIs7 (nape-1::mCherry unc-25::mrfp)* | 14 | 104 (2) | ns | - |
|  | *jluIs2 (nape-2::gfp unc-25::mrfp)* | 16 | 97 (4) | <0.001 | - |
|  | *daf-2(e1368)* | 26 | 97 (8) | < 0.0001 | - |
|  | *daf-2(e1368); jluIs7 (nape-1::mCherry unc-25::mrfp)* | 22 | 102 (3) | < 0.0001 | < 0.0001 |
|  | *daf-2(e1368); jluIs2 (nape-2::gfp unc-25::mrfp*) | 26 | 93 (6) | < 0.0001 | ns |
| ***Trial 2*** | N2 | 15 | 98 (6) | - | - |
|  | *jluIs7 (nape-1::mCherry unc-25::mrfp)* | 12 | 86 (19) | <0.05 | - |
|  | *jluIs2 (nape-2::gfp unc-25::mrfp)* | 15 | 100 (8) | ns | - |
|  | *daf-2(e1368)* | 26 | 92 (9) | < 0.0001 | - |
|  | *daf-2(e1368); jluIs7 (nape-1::mCherry unc-25::mrfp)* | 22 | 97 (4) | < 0.0001 | <0.001 |
|  | *daf-2(e1368); jluIs2 (nape-2::gfp unc-25::mrfp*) | 26 | 70 (28) | < 0.0001 | ns |
| ***Trial 3**** | N2 | 14 | 94 (3) | - | - |
|  | *jluIs7 (nape-1::mCherry unc-25::mrfp)* | 12 | 95 (5) | <0.001 | - |
|  | *jluIs2 (nape-2::gfp unc-25::mrfp)* | 16 | 81 (10) | ns | - |
|  | *daf-2(e1368)* | 25 | 93 (11) | < 0.0001 | - |
|  | *daf-2(e1368); jluIs7 (nape-1::mCherry unc-25::mrfp)* | 21 | 90 (11) | < 0.0001 | <0.0001 |
|  | *daf-2(e1368); jluIs2 (nape-2::gfp unc-25::mrfp)* | 25 | 86 (20) | < 0.00001 | ns |

* Data shown in Figure 6B
