# Supplementary material for: Characterization of N-Acyl Phosphatidylethanolamine-Specific Phospholipase-D Isoforms in the Nematode Caenorhabditis elegans
Source: PLoS One. 2014 Nov 25;9(11):e113007. doi: 10.1371/journal.pone.0113007 (PMC4244089; doi:10.1371/journal.pone.0113007)

**Figure S8: *nape-1::mCherry* and *nape-2::gfp* expression is not affected by changes in growth temperature.** Confocal images of *nape-1(OE)* and *nape-2(OE)* worms grown at **(A)** 15^o^C and **(B)** 25^o^C. The dashed lines in the *nape-1(OE)* images indicate the *unc-25::mrfp* co-injection marker.

**A 15°C**

NAPE-1::mCherry NAPE-2::GFP


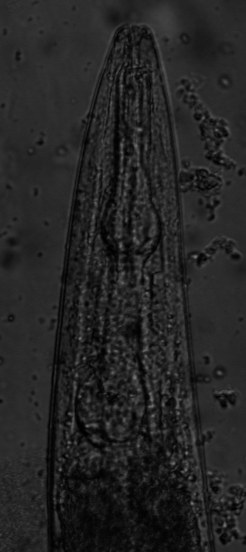

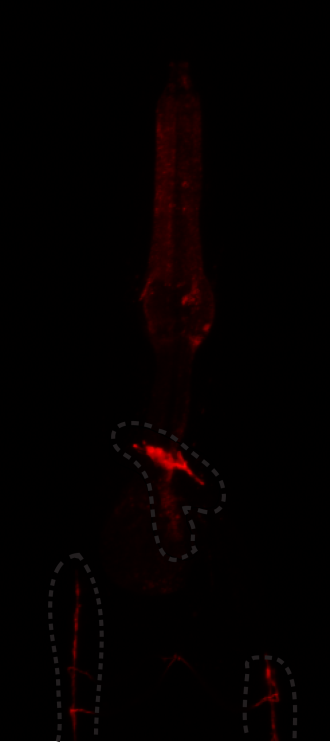

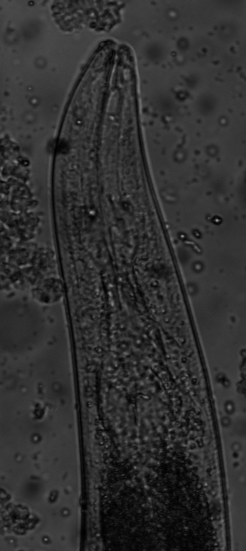

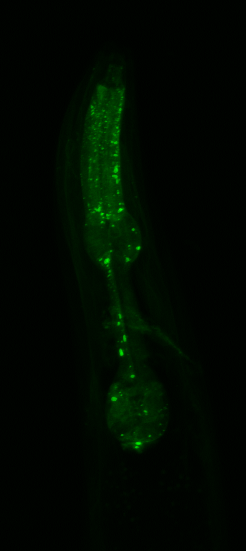


**B 25°C**

NAPE-1::mCherry NAPE-2::GFP


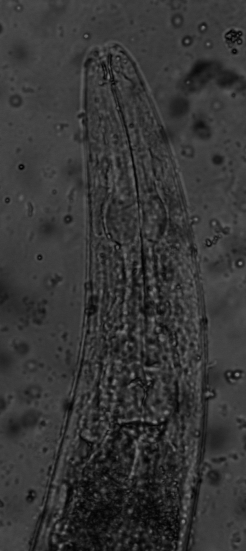

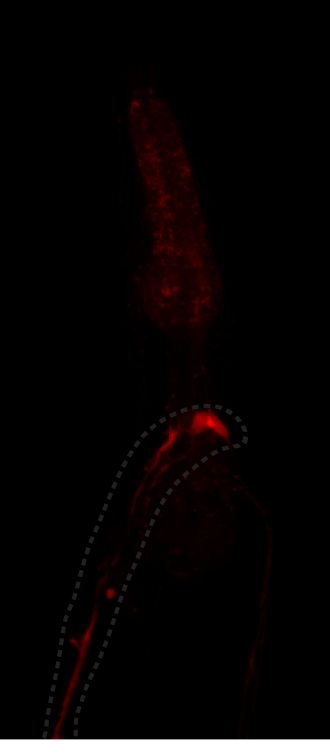

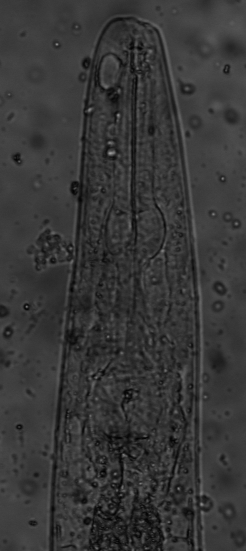

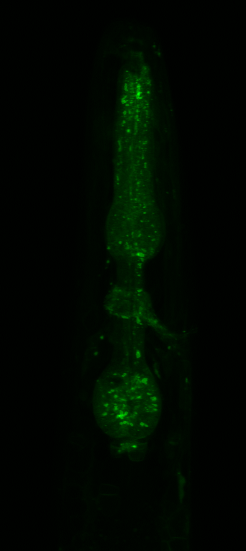

Supplement: Figure S8 — nape-1::mCherry and nape-2::gfp expression is not affected by changes in growth temperature. Confocal images of nape-1(OE) and nape-2(OE) worms grown at (A) 15°C and (B) 25°C. The dashed lines in the nape-1(OE) images indicate the unc-25::mrfp co-injection marker. (DOCX) [file pone.0113007.s008.docx]
