# Supplementary material for: Characterization of N-Acyl Phosphatidylethanolamine-Specific Phospholipase-D Isoforms in the Nematode Caenorhabditis elegans
Source: PLoS One. 2014 Nov 25;9(11):e113007. doi: 10.1371/journal.pone.0113007 (PMC4244089; doi:10.1371/journal.pone.0113007)
Supplement: Table S1 — Raw growth data for Figures 2A–C , 3A–C , 4A&B and 5A–C . (DOCX) [file pone.0113007.s009.docx]

**Table S1: Raw growth data for Figures 2A-C, 3A-C, 4A&B and 5A-C.**

| **Figure** | **Temp** | **Strain** | **Raw data (% greater than L4 stage)** | **Mean ± sd** |
| --- | --- | --- | --- | --- |
| 2A | 15°C | N2 | 99, 100, 100, 99, 96, 100 | 99±1.5 |
|  |  | *nape-1(tm3860)* | 100, 97, 99, 100, 100, 100 | 99±1.2 |
|  |  | *nape-2(tm6254)* | 100, 99, 100, 100, 100 | 100±0.4 |
| 2B | 20°C | N2 | 100, 100, 100 | 100 |
|  |  | *nape-1(tm3860)* | 99, 100, 100 | 100±0.9 |
|  |  | *nape-2(tm6254)* | 98, 99, 100 | 99±0.5 |
| 2C | 25°C | N2 | 100, 100, 100, 99, 99 | 100±0.6 |
|  |  | *nape-1(tm3860)* | 100, 99, 100, 99, 100 | 100±0.6 |
|  |  | *nape-2(tm6254)* | 97, 89, 96, 100, 97 | 96±4 |
| 3A | 15°C | N2 | 98, 98, 99, 97, 100, 100, 98, 98, 100 | 99±1.1 |
|  |  | *nape-1(OE)* | 92, 87, 85, 90, 91, 91, 89, 84, 87 | 87±3.3 |
|  |  | *nape-2(OE)* | 5, 19, 46, 22, 8, 63, 78, 59, 35 | 37±3 |
|  |  | *nape-1(OE); nape-2(OE)* | 64, 66, 69, 53 | 63±7 |
| 3B | 20°C | N2 | 100, 100, 100, 100 | 100 |
|  |  | *nape-1(OE)* | 92, 97, 89, 92 | 92±3.5 |
|  |  | *nape-2(OE)* | 80, 94, 73, 82 | 82±8.8 |
|  |  | *nape-1(OE); nape-2(OE)* | 72, 91, 84, 72 | 80±9.5 |
| 3C | 25°C | N2 | 98, 99, 99, 99, 99, 100, 99, 100, 100 | 99±0.6 |
|  |  | *nape-1(OE)* | 42, 46, 57, 9, 8, 4, 9, 10, 11 | 21±20 |
|  |  | *nape-2(OE)* | 22, 46, 82, 86, 87, 77, 87, 79, 82 | 72±23 |
|  |  | *nape-1(OE); nape-2(OE)* | 16, 44, 59, 3 | 31±26 |
| 4A | 15°C | N2† | 98, 98, 99, 97, 100, 100, 98, 98, 100 | 99±1.1 |
|  |  | *faah-1(tm5011)* | 100, 100, 100, 98, 100 | 100±0.9 |
|  |  | *nape-1(OE)* † | 92, 87, 85, 90, 91, 91, 89, 84, 87 | 87±3.3 |
|  |  | *faah-1(tm5011); nape-1(OE)* | 77, 61, 74, 73, 67 | 70±6.4 |
|  | 25°C | N2‡ | 98, 99, 99, 99, 99, 100, 99, 100, 100 | 99±0.6 |
|  |  | *faah-1(tm5011)* | 99, 100, 100, 100, 98 | 99±0.9 |
|  |  | *nape-1(OE)* ‡ | 42, 46, 57, 9, 8, 4, 9, 10, 11 | 21±20 |
|  |  | *faah-1(tm5011); nape-1(OE)* | 0, 1, 3, 0, 0 | 0.8±1.3 |
| 4B | 15°C | N2† | 98, 98, 99, 97, 100, 100, 98, 98, 100 | 99±1.1 |
|  |  | *faah-1(tm5011)* | 100, 100, 100, 98, 100 | 100±0.9 |
|  |  | *nape-2(OE)* † | 5, 19, 46, 22, 8, 63, 78, 59, 35 | 37±3 |
|  |  | *faah-1(tm5011); nape-2(OE)* | 99, 95, 95, 97, 97 | 97±1.7 |
|  | 25°C | N2‡ | 98, 99, 99, 99, 99, 100, 99, 100, 100 | 99±0.6 |
|  |  | *faah-1(tm5011)* | 99, 100, 100, 100, 98 | 99±0.9 |
|  |  | *nape-2(OE)* ‡ | 22, 46, 82, 86, 87, 77, 87, 79, 82 | 72±23 |
|  |  | *faah-1(tm5011); nape-2(OE)* | 98, 94, 92, 94, 98 | 95±2.7 |
| 5A | 15°C | N2 | 100, 100, 100, 100 | 100 |
|  |  | *nape-1(OE)* | 93, 90, 89, 94 | 93±3 |
|  |  | *nape-2(OE)* | 100, 86, 66, 70 | 81±16 |
|  |  | *fat-1(ok2323)* | 100, 100, 100, 100 | 100 |
|  |  | *fat-1(ok2323); nape-1(OE)* | 90, 79, 86, 91 | 87±5.8 |
|  |  | *fat-1(ok2323); nape-2(OE)* | 97, 73, 71, 51 | 73±19 |
|  |  | *fat-4(ok958)* | 67, 33, 65, 38 | 51±18 |
|  |  | *fat-4(ok958); nape-1(OE)* | 11, 53, 3, 8 | 19±23 |
|  |  | *fat-4(ok958); nape-2(OE)* | 14, 35, 7, 13 | 17±12 |
|  |  | *fat-3(wa22)* | 79, 9, 0, 0 | 22±38 |
|  |  | *fat-3(wa22); nape-1(OE)* | 0, 4, 0, 50 | 14±25 |
|  |  | *fat-3(wa22); nape-2(OE)* | 0, 0, 0, 0 | 0 |
| 5B | 20°C | N2 | 100, 100, 100, 100 | 100 |
|  |  | *nape-1(OE)* | 100, 97, 97, 94 | 97±2.4 |
|  |  | *nape-2(OE)* | 100, 86, 84, 76 | 87±10 |
|  |  | *fat-1(ok2323)* | 100, 100, 100, 100 | 100 |
|  |  | *fat-1(ok2323); nape-1(OE)* | 98, 92, 95, 86 | 93±5.1 |
|  |  | *fat-1(ok2323); nape-2(OE)* | 100, 90, 74, 87 | 88±11 |
|  |  | *fat-4(ok958)* | 100, 100, 100, 100 | 100 |
|  |  | *fat-4(ok958); nape-1(OE)* | 51, 43, 47, 31 | 43±8.4 |
|  |  | *fat-4(ok958); nape-2(OE)* | 82, 59, 23, 50 | 54±25 |
|  |  | *fat-3(wa22)* | 100, 80, 100, 94 | 93±9.3 |
|  |  | *fat-3(wa22); nape-1(OE)* | 50, 73, 52, 32 | 51±17 |
|  |  | *fat-3(wa22); nape-2(OE)* | 71, 69, 71, 67 | 69±1.9 |
| 5C | 25°C | N2 | 100, 98, 100, 100, 100 | 100±0.9 |
|  |  | *nape-1(OE)* | 55, 56, 51, 34, 40 | 47±10 |
|  |  | *nape-2(OE)* | 94, 66, 69, 59, 71 | 72±13 |
|  |  | *fat-1(ok2323)* | 100, 100, 100, 99, 100 | 100±0.3 |
|  |  | *fat-1(ok2323); nape-1(OE)* | 45, 15, 16, 8, 26 | 22±14 |
|  |  | *fat-1(ok2323); nape-2(OE)* | 94, 57, 45, 37, 47 | 56±23 |
|  |  | *fat-4(ok958)* | 91, 100, 93, 63, 100 | 90±15 |
|  |  | *fat-4(ok958); nape-1(OE)* | 8, 2, 5, 0, 8 | 4±3.5 |
|  |  | *fat-4(ok958); nape-2(OE)* | 29, 14, 24, 17, 32 | 23±7.4 |
|  |  | *fat-3(wa22)* | 92, 85, 68, 70, 87 | 80±11 |
|  |  | *fat-3(wa22); nape-1(OE)* | 9, 14, 3, 47 | 18±19 |
|  |  | *fat-3(wa22); nape-2(OE)* | 38, 43, 26, 9, 13 | 26±15 |

† Data same as Fig 3A

‡ Data same as Fig 3C
